# Tracing the disc: The novel qualitative morphometric MRI based disc degeneration classification system

**DOI:** 10.1002/jsp2.1321

**Published:** 2024-03-18

**Authors:** Zafer Soydan, Emru Bayramoglu, Devrim Ulas Urut, Ahmet Celal Iplikcioglu, Cengiz Sen

**Affiliations:** ^1^ BHT Clinic İstanbul Tema Hospital, Department of Orthopedics and Traumatology Nisantası University Istanbul Turkey; ^2^ Department of Orthopedics and Traumatology Bursa City Hospital Bursa Turkey; ^3^ BHT Clinic İstanbul Tema Hospital Department of Radiology Istanbul Turkey; ^4^ BHT Clinic İstanbul Tema Hospital Department of Neurochirurgie Istanbul Turkey; ^5^ İstanbul University Department of Orthopedics And Traumatology Istanbul Turkey

**Keywords:** disc degeneration, MRI, novel morphometric classification, reliability tests

## Abstract

**Background:**

This study aimed to develop a classification system for lumbar disc degeneration using routine magnetic resonance images (MRIs) that is easily applicable and unaffected by existing classifications' limitations, and to compare its reliability, reproducibility, and discriminative power to the widely used Pfirrmann classification.

**Methods:**

Five features were graded. This new classification system has eight grades, with at least one of these five features altering each grade. The T2‐weighted sagittal images were acquired using a rapid spin‐echo sequence with a repetition time of 2680 to 4900 milliseconds, an echo time of 100 to 109 milliseconds, and an echo train length of 17. Slice thick was 4 mm and the display field of view was 32 × 32 cm. The new classification system used five features: signal intensity, disc height, disc boundary regularity, and nucleus annulus separation. Increased signal intensity, decreased height, decreased regularity, and decreased nucleus‐annulus separation indicated degeneration. Four raters classified 400 discs from 80 patients using the Pfirrmann and Novel systems. Statistical analyses were conducted to investigate reliability and correlation.

**Results:**

The overall ICC and kappa values were found to be higher in the novel classification. (0.988 indicating excellent agreement for ICC and 0.76/0.94 indicating good–very good agreement for kappa). The Kendall tau *c* value, which shows the correlation between the two classifications and indicates the validity of the new classification, was 0.872, which is very strong. Through the use of cross‐tabulations, the discriminatory power of the two newly added classification criteria was determined.

**Conclusions:**

This study demonstrates the intra‐rater and inter‐rater reliability of an easy‐to‐use, discriminative novel morphometric MRI based classification system for lumbar disc degeneration. The differentiation of grades based on five distinct criteria may generate novel hypotheses regarding treatment selection and response monitoring, as well as new insights into the study of disc degeneration.

## Graphical abstract

The novel and easy to applicable magnetic resonance image based disc degeneration classification system. We think it addresses the shortcomings of existing classification systems.

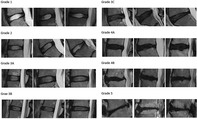

## 1 İNTRODUCTION

Low back pain (LBP) is a significant neurological condition that affects over 540 million individuals globally.^1^ Even though LBP affects all age groups, its frequency increases with age and is more prevalent in women than in men. Several factors contribute to LBP, but structural and functional defects in the intervertebral disc in the spine, which result in its degeneration, are among the most significant.^2^, ^3^ The degenerated disc is characterized by dehydration of extracellular matrix (ECM), decreased disc height, hypocellularity and change in the phenotype of nucleus pulposus (NP) cells.^4^ Despite being primary causes of disability, disc degeneration and associated LBP are rarely treated.^5^ Current remedies are predominantly palliative and primarily focused on alleviating painful symptoms as opposed to addressing the underlying causes. In severe cases, spinal fusion of the vertebral bodies is the standard treatment for alleviating symptoms. However, the restriction of spinal motion and altered loading biomechanics that may accompany this treatment may result in disc degeneration in adjacent segments, requiring reoperation.^6^, ^7^ The ideal treatment would simultaneously alleviate agonizing symptoms and restore disc structure and function by addressing the underlying molecular or biological causes of disc degeneration. Recently, regenerative therapies, including enzyme treatments, monoclonal antibodies, and cell‐based therapies with the potential to be applied in the early stages of disc degeneration, have become increasingly prominent.^8^, ^9^

Chondolyase injection may be associated with increased degeneration in initially Pfirrmann^10^ grade 2 or 3 discs, according to an investigation into chemonucleolysis treatment‐related factors that may contribute to disc degeneration.^11^ An additional study conducted by the same author documented that chemonucleolysis treatment led to Pfirmann grade progression in 61.5% of patients within 3 months.^12^ An elucidation of this relationship could be achieved through the implementation of a more precise classification.

In their research, Zhu et al. found that the administration of human growth and differentiation factor 5 (rhGDF5) therapy effectively mitigated disc degeneration in rodents.^13^ The GDF5 gene variation has been demonstrated to be associated with degenerative disc degeneration in humans.^14^ The involvement of interleukin 1 (IL‐1) and tumor necrosis factor‐alpha (TNFA) cytokines in DDD has been documented. Monoclonal antibody therapies have been developed to alter the activity of these cytokines.^15^ Nucleus pulposus progenitor cells were recognized by Sakai et al. as a beneficial therapeutic approach exemplifying cell‐based therapies. Both morphological and magnetic resonance imaging (MRI) staging demonstrated that tie‐2+ NP cells are diminished during disc degeneration, as demonstrated by Sakai et al. Cell‐based therapies for the treatment of disc degeneration in both animals and humans have demonstrated encouraging results.^16^, ^17^, ^18^

Degeneration grading in greater detail may provide insight into the identification of the NP lineage cell differentiation pathway.

Better imaging and evaluation of the degeneration are essential for determining which treatment is most suitable for which patient and for choosing the correct treatment.^19^ Several limitations of morphologically based classifications, such as Pfirrmann's, were identified by Nagy et al. It has been documented that variations in visual perception, particularly when observing subtle changes, can result in significant discrepancies between observers; this discrepancy has consistently been linked to an overestimation or underestimation of the grading boundary.^20^ By refining and diversifying grading boundaries, this estimation limitation may be eliminated. Insufficient sensitivity has also been attributed to this classification in evaluating initial biochemical alterations. A novel grading, which incorporates early modifications, might thus assist in resolving this restriction.^21^

The Pfirrman classification is the morphologic‐qualitative classification which is most frequently applied. The degenerative process is categorized into five distinct grades. For interrater and intrarater reliability, Carrino et al. reported kappa values ranging from 0.66 to 0.74, whereas Urrutia et al. discovered the same values at 0.83 to 0.89.^22^, ^23^ The investigation utilized images acquired through a 9 T MRI device and determined both values to be 0.91.^24^

The 5‐stage Pfirrmann classification was reported that high intra‐class differences and low inter‐class differences reduced efficiency and it has been criticized for providing only non‐discriminative information.^25^ Modified morphological^26^ and quantitative^21^, ^25^, ^27^, ^28^ classification systems have been devised in order to surmount these constraints. In addition, despite the success of quantitative classifications, there are difficulties encountered in practice, such as the need for expensive software and specialized equipment, lengthy examination periods, low sensitivity, and standardization problems. Recently, classification systems utilizing artificial intelligence (AI) networks have been devised, and these studies have reported varying degrees of reproducibility. Existing AI solutions are not new classifications but rather efforts to improve the performance of existing classification systems. Given the findings of these studies, it is our conviction that a classification system is required that exhibits greater discriminatory power than the current classification systems and is both readily obtainable and interpretable. In this study, we intend to publish the results of a new grading method we developed using T2W sagittal slices, which are easily applicable, obtained by default from every MRI machines, and unaffected by the limitations of existing morphometric and quantitative classifications.

## 2 MATERIALS AND METHODS

The efficacy of artificial intelligence networks in classifying disc degeneration and their influence on clinicians' decision‐making was the subject of a published study.^29^ The author was motivated to devise a new classification system as a result of the observable limitations that were believed to impact performance during this study.

### 2.1 Subject data

All procedures performed in studies involving human participants were in accordance with the ethical standards of the Ethics Committee of Kuopio University Hospital and with the 1964 Helsinki declaration and its later amendments or comparable ethical standards. Institutional Review Board of Nisantasi University (Protocol No. 2022/46). Four hundred consecutive intervertebral discs from L1–L2 to L5–S1 on lumbar spine MRI examinations of 80 subjects (40 men and 40 women) with a mean age of 43 ± 9 years (range, 18–77 years) who presented to our clinic with low back pain between January 2023 and July 2023 were utilized in this study. Midsagittal T2W sections were obtained from each patient and anonymized. A 1.5‐T scanner was used to do MRIs of the lumbar spine (GE, Signa, 1.5‐T). Only sagittal T2W images were utilized in this study. T2W sagittal images were acquired using a fast spin‐echo sequence with a repetition time of 2680–4900 msec, an echo time of 100–109 msec, and an echo train length of 17. DFOV was 32 × 32 cm, with a slice thickness of 4 mm. The senior author formulated a comprehensive grading system for lumbar disc degeneration based on these criteria given in Table 1.

**TABLE 1:** Classification of disc degeneration, each criterion is given in a separate column.

Grade	Structure	Np‐Af distinction	Signal intensity	Ivd height	Disc border regularity
1	Homogenous	Clear	Hyperintense—CSF	Normal	Normal
2	Homogenous	Clear	Hyperintense to VB	Normal	Normal
3A	Largely Homogenous (HGB‐)	Clear	Hypointense to VB/Gray	Normal	Normal
3B	İnhomogenous (HGB+)	Clear	Hypointense to VB / Gray	Normal	Normal
3C	İnhomogenous	Unclear	Dark gray	Slight decrease (esp. post)	Normal
4A	İnhomogenous	Lost	Black disc	Decreased less than half	Partially regular
4B	İnhomogenous	Lost	Black disc	Decreased more than half	Irregular/bumpy
5	İnhomogenous	Lost	Black disc	Collapsed/body contact	Deteriorated

Abbreviations: CSF, cerebrospinal fluid; HGB: horizontal gray bands; VB: vertebral body.

#### 2.1.1 Definitions

A loss of disc signal on a T2W image is indicative of decreased proteoglycans and water content in the disc.^30^ Depending on the stage of degeneration, MRI can reveal structural alterations in the disc and height loss.^31^ As disc degeneration progresses, it is known that both the demarcation of the annulus fibers deteriorates and the disc height decreases.^26^ The novel classification system was based on the evaluation of five features: disc homogeneity, nucleus annulus separation, signal intensity, disc height and regularity of disc borders. Each class differentiated from the previous class by at least one feature. Although some of these criteria are also used in the well‐known Pffirmann classification and later Griffith modification, our study has major differences from both of these. First, Pfirrmann used only CSF intensity as a criterion for evaluating signal intensity for the onset of degeneration, whereas in our classification, both CSF and vertebral body signal intensity were taken into account. Second, unlike Pfirrmann and Griffith, the irregularity of the disc boundaries, which we observed in the late grades of disc degeneration, was also taken into account as a separate criterion for the first time. In the Griffith modification, pre sacral fat was used in addition to CSF for signal intensity evaluation. However, disc height was used as the only discriminating feature in the last four stages of the degeneration process which they divided into eight stages and no discrimination was defined in terms of the other two features which they used.

Although most authors regard an intranuclear cleft to be a normal finding in the aging process, there are studies that suggest it is a pre‐degenerative lesion. Theo et al. opined that cleft is a pre‐degenerative lesion and that decreased osmotic pressure is associated with cleft formation.^32^ In a separate study, the same authors demonstrated that the cleft in MRI sections corresponded to the multidirectional cleavages observed in histological sections of the nucleus pulposus.^33^ We therefore deemed cleft a pre‐degenerative lesion in our system. A comparative analysis of the decisive grades of the criteria associated with both classifications is presented in Table 2.

**TABLE 2:** Comparison of the overlapping and diverging criteria between the Novel and Pfirmann classifications. Vertebral body intensity and disc border irregularity criteria of the novel classification provide discrimination between the two classifications. Each criterion and the grades in which it shows distinctive features are given in brackets.

	Novel	Pfirrmann
Discriminative criteria		
Disc Homogeneity	Homogen: 1,2,3a	Homogen: 1
Nucleus Annulus Separation	Clear	Clear
	1,2,3a,3b	1,2
Signal intensity	CSF(1)	CSF(1, 2)
	VB (2,3a)	VB−
Disc height	4a,4b,5	4,5
Disc border irregularity	4b	‐

Abbreviations: CSF, cerebrospinal fluid; VB: vertebral body.

## 3 İMAGE ASSESSMENT

### 3.1 Grading by Pfirrmann System

Four raters (two senior orthopaedists, one muscoluskeletal senior radiologist and one experienced neurosurgeon which all are more than 15 years of experience) classified the images two times with an interval of 4 weeks according to Pfirrmann Classification. The data set was randomized before each session.

### 3.2 Grading by novel system

**FIGURE 1:**
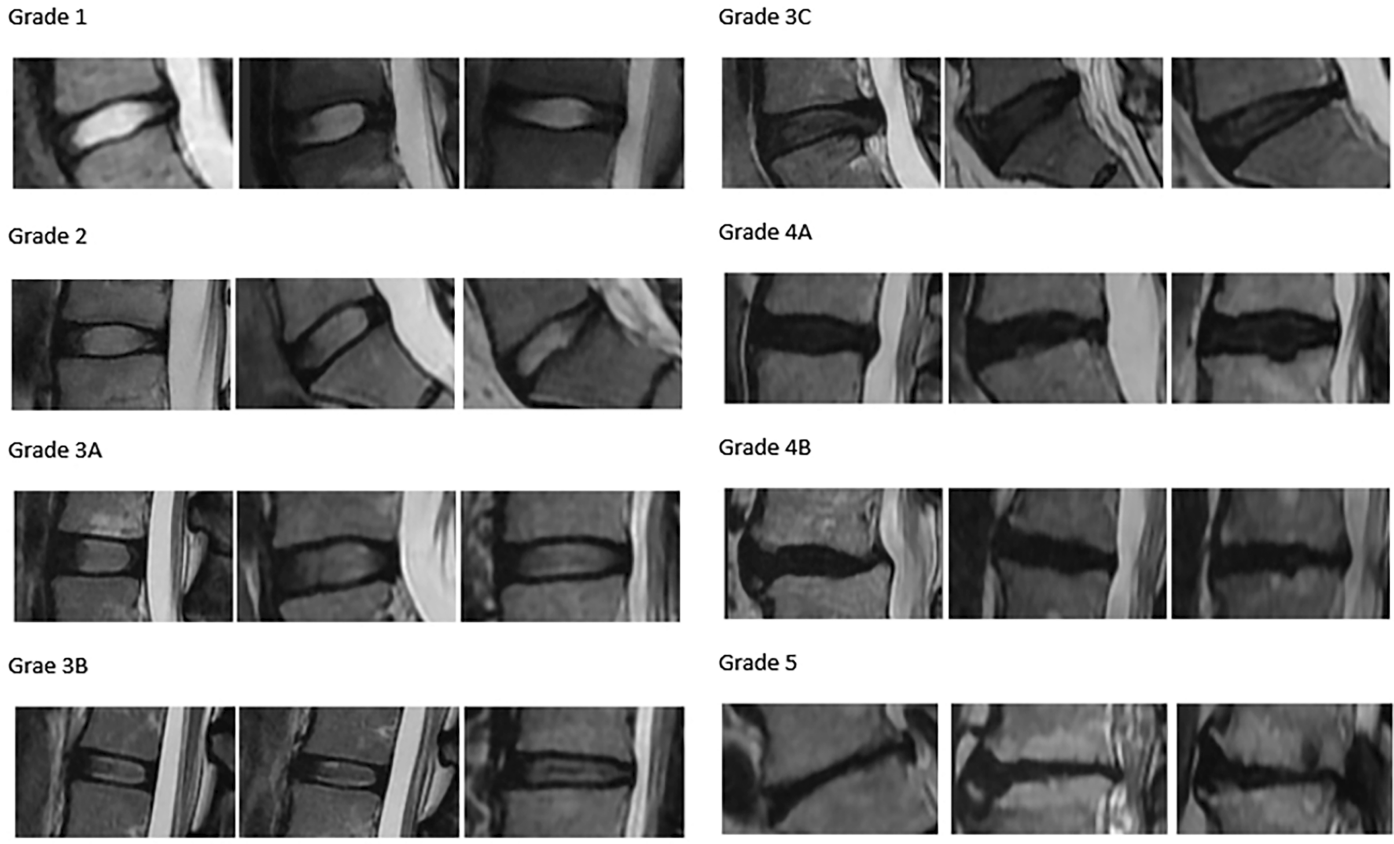
Novel method of classification of eight stages of disc degeneration.

While one of the two orthopaedists designed the novel classification system, the other raters were trained using the image reference chart and the explanatory annotations, including a flowchart (Figure 1, Chart 1; Supporting information, Figure 2). No face‐to‐face training was provided. Same four raters also classified the images according to novel classification. The data set was randomized before each session. The term ground truth (GT) refers to the grade that is regarded by all as being completely accurate for each disc. For both classification systems, the same method was used to determine GT. The three consistent grading outcomes of the four raters were adopted as GT. In the absence of a quorum, the discs were re‐evaluated by four physicians in a separate session and labeled by consensus.

**FIGURE 2:**
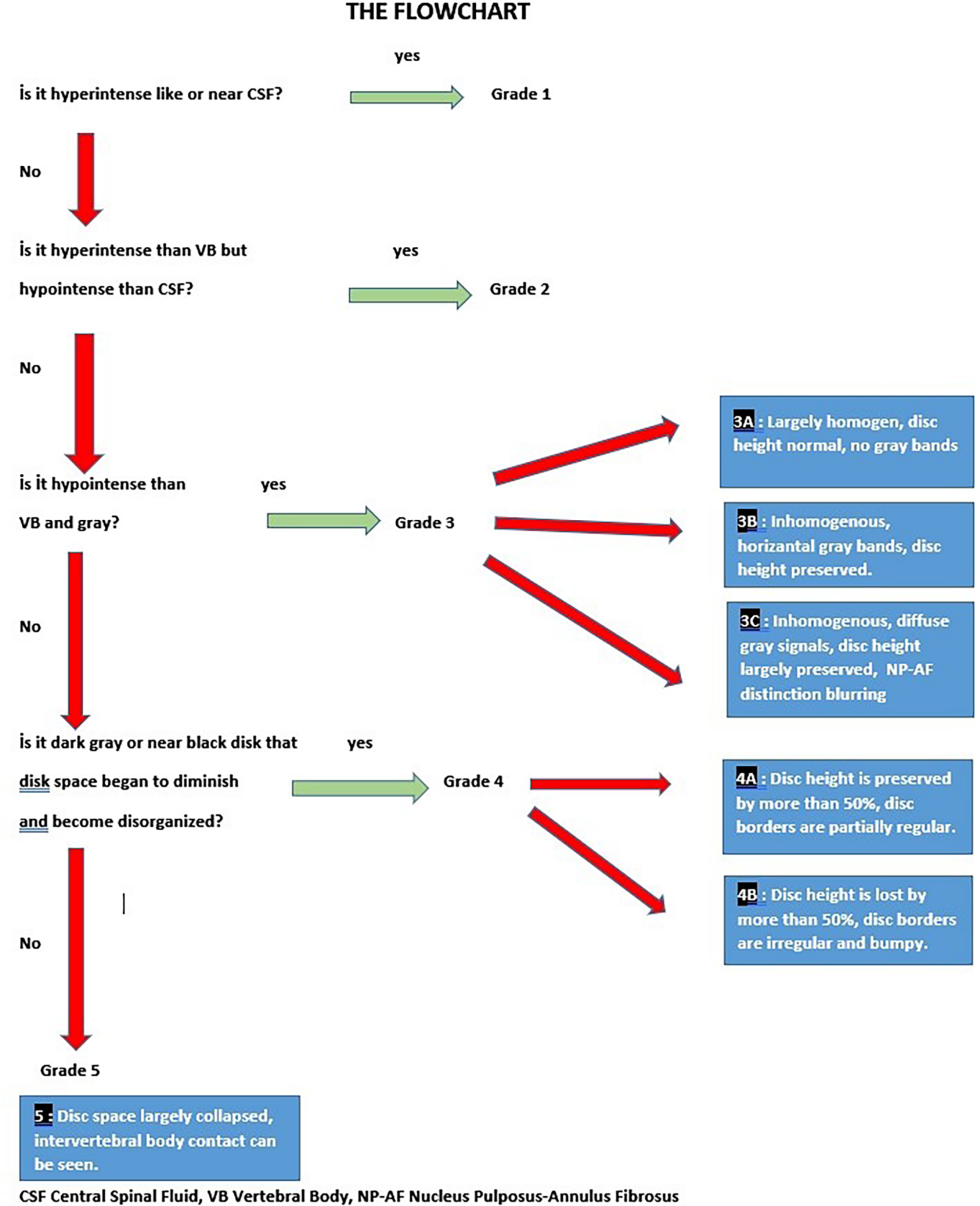
The flowchart of classification.

## 4 STATISTICS

In data analysis, crosstabs, Kendall tau‐c coefficients and intraclass correlation coefficients (ICC) and kappa values for inter‐rater and intra‐rater reliability were used. *p* < 0.05 level was taken into consideration for statistical significance, and the analyses were performed using the SPSS (version 25) package program. After the data were collected within the scope of the research, post‐hoc power analysis was performed. The conditions determined for power analysis were an alpha level of 0.05, a medium effect size, and a two‐way hypothesis. Power analysis was performed through the Gpower (version 3.1.9.7) package program. As a result of the power analysis, it was determined that 1‐beta = 0.82, and it was determined that the research had acceptable power.

## 5 RESULTS

Four hundred discs from 80 consecutive patients were reviewed. The novel system has greater ICC values (0.988 vs 0.979). İnterobserver and intraobserver reliability tests of both systems is given in Table 3.

**TABLE 3:** The new system has greater intra and inter‐rater reliability than the Pfirrmann classification.

		Novel System								Pfirrmann							
		R1		R2		R3		R4		R1		R2		R3		R4	
Reliability	Disc	ICC	Kappa	ICC	Kappa	ICC	Kappa	ICC	Kappa	ICC	Kappa	ICC	Kappa	ICC	Kappa	ICC	Kappa
Intra‐rater	L	0.990	0.940	0.948	0.760	0.983	0.899	0.980	0.864	0.957	0.792	0.939	0.955	0.930	0.698	0.987	0.939
İnter‐rater (ICC)	L	0.988								0.979							

*Note*: ICC values: <0.5 is low; 0.5–0.75 moderate; 0.75–0.90 high; >0.9 indicates very high reliability. Kappa values: <0.40 is low; 0.41–0.60 moderate; 0.61–0.80 high; >0.81 very high reliability.

Abbreviation: R, rater.

Overall ICC and kappa values were found to be higher in the novel system. The Tau‐c results of the raters ranged between 0.864 and 0.918. When comparing the GT results of the two classifications, the overall tau‐c coefficient was determined to be 0.872. All of the classifications made by the four experts based on the old and new classifications have correlation values of 0.80 or higher. A correlation coefficient of 0.80 or greater indicates that the two variables whose relationship is being examined are remarkably similar. The correlation analysis between the two systems is given in Table 4.

**TABLE 4:** Analyzing the correlation between the two systems using Kendall Tau. The results of the four raters and GT for each disc are presented.

	R1	R2	R3	R4	GT
Disc	Tau‐c	Tau‐c	Tau‐c	Tau‐c	Tau‐c
L1	0.854**	0.825**	0.884**	0.880**	0.824**
L2	0.908**	0.853**	0.880**	0.938**	0.886**
L3	0.918**	0.873**	0.900**	0.923**.	0.896**
L4	0.878**	0.868**	0.843**	0.915**	0.866**
L5	0.889**	0.844**	0.882**	0.895**	0.856**
L	0.897**	0.864**	0.888**	0.918**	0.872**

** Tau‐c > 0.80 indicates that the two variables whose relationship is being examined are remarkably similar.

A cross‐tab comparison was performed to ascertain and compare the distribution of discs in both classification systems according to their GT. The outcomes are presented in Table 5.

**TABLE 5:** Crosstable between the classifications according to their GT.

			Pfirrmann					Total
			1	2	3	4	5	
Novel System	1	Count	4	1	0	0	0	5
		% within Novel	80.0%	20.0%	0.0%	0.0%	0.0%	100.0%
		% within Pfirrmann	9.5%	1.1%	0.0%	0.0%	0.0%	1.3%
		% of Total	1.0%	0.3%	0.0%	0.0%	0.0%	1.3%
	2	Count	36	40	2	0	0	78
		% within Novel	46.2%	51.3%	2.6%	0.0%	0.0%	100.0%
		% within Pfirrmann	85.7%	43.5%	2.7%	0.0%	0.0%	19.5%
		% of Total	9.0%	10.0%	0.5%	0.0%	0.0%	19.5%
	3A	Count	2	21	23	0	0	46
		% within Novel	4.3%	45.7%	50.0%	0.0%	0.0%	100.0%
		% within Pfirrmann	4.8%	22.8%	31.1%	0.0%	0.0%	11.5%
		% of Total	0.5%	5.3%	5.8%	0.0%	0.0%	11.5%
	3B	Count	0	30	29	2	0	61
		% within Novel	0.0%	49.2%	47.5%	3.3%	0.0%	100.0%
		% within Pfirrmann	0.0%	32.6%	39.2%	2.0%	0.0%	15.3%
		% of Total	0.0%	7.5%	7.2%	0.5%	0.0%	15.3%
	3C	Count	0	0	14	14	0	28
		% within Novel	0.0%	0.0%	50.0%	50.0%	0.0%	100.0%
		% within Pfirrmann	0.0%	0.0%	18.9%	14.1%	0.0%	7.0%
		% of Total	0.0%	0.0%	3.5%	3.5%	0.0%	7.0%
	4A	Count	0	0	4	47	2	53
		% within Novel	0.0%	0.0%	7.5%	88.7%	3.8%	100.0%
		% within Pfirrmann	0.0%	0.0%	5.4%	47.5%	2.2%	13.3%
		% of Total	0.0%	0.0%	1.0%	11.8%	0.5%	13.3%
	4B	Count	0	0	2	31	14	47
		% within Novel	0.0%	0.0%	4.3%	66.0%	29.8%	100.0%
		% within Pfirrmann	0.0%	0.0%	2.7%	31.3%	15.1%	11.8%
		% of Total	0.0%	0.0%	0.5%	7.8%	3.5%	11.8%
	5	Count	0	0	0	5	77	82
		% within Novel	0.0%	0.0%	0.0%	6.1%	93.9%	100.0%
		% within Pfirrmann	0.0%	0.0%	0.0%	5.1%	82.8%	20.5%
		% of Total	0.0%	0.0%	0.0%	1.3%	19.3%	20.5%
Total		Count	42	92	74	99	93	400
		% within Novel	10.5%	23.0%	18.5%	24.8%	23.3%	100.0%
		% within Pfirrmann	100.0%	100.0%	100.0%	100.0%	100.0%	100.0%
		% of Total	10.5%	23.0%	18.5%	24.8%	23.3%	100.0%

*Note*: The colours indicate the common distribution divisions between the classification systems.

**FIGURE 3:**
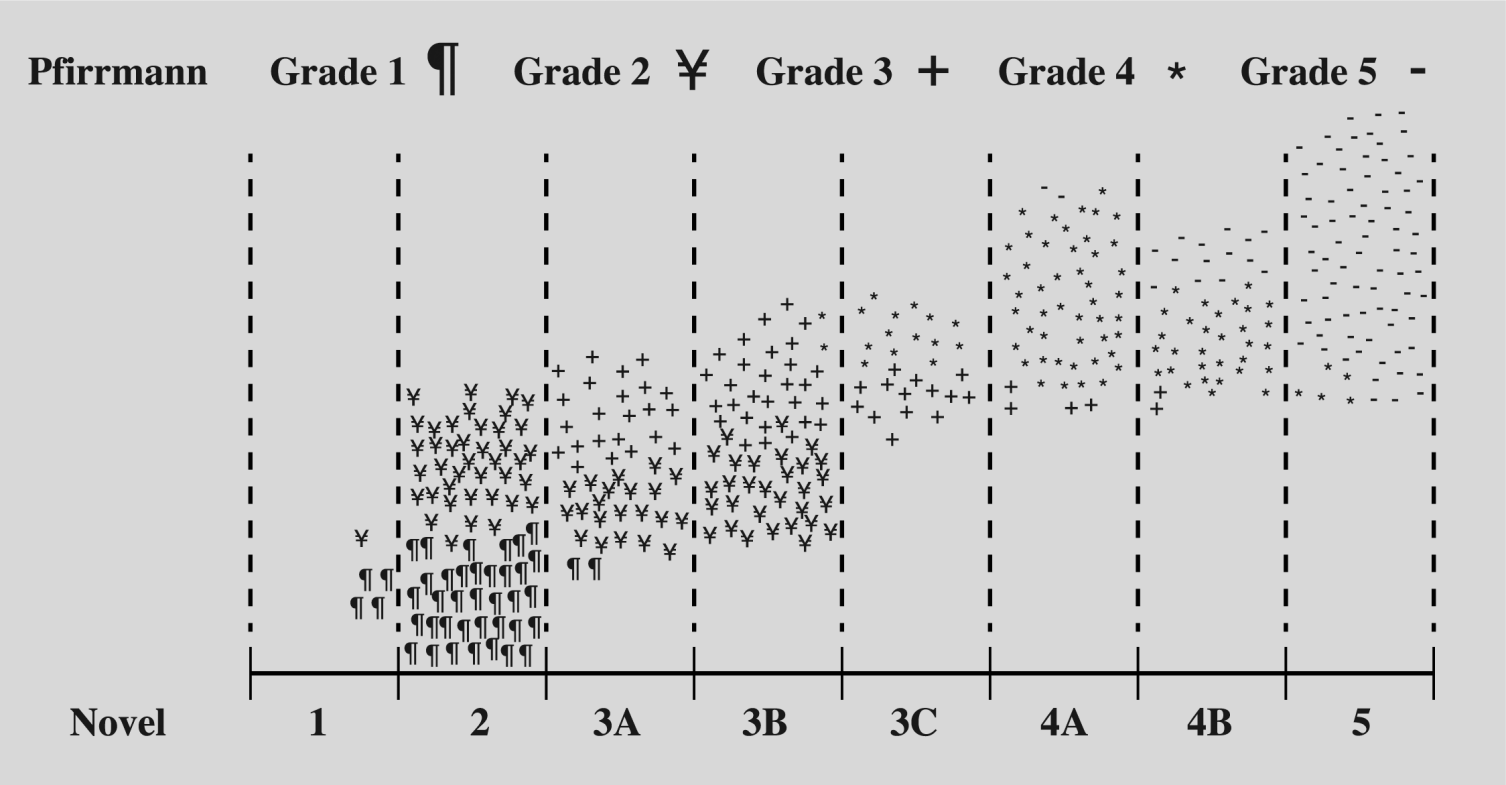
Comparative distribution between Pfirrmann and novel system. Each Pfirrmann grade is represented by a different symbol. Each point corresponds to a disc, and a total of 400 discs are distributed across the grades of novel classification.

The discriminative distribution between the Pfirrmann classification and the new classification is given in Figure 3.

## 6 DISCUSSION

Pfirrmann's classification is the most widespread morphologic classification used in IVDD grading. However, this classification has been documented to have several limitations. In particular, it is not adequate discriminative in older subjects; the features described and the images provided may contribute to confusion regarding the level of disc degeneration, and visual difficulties in T2W signal changes may result in differences between observers with varying levels of experience.^20^, ^26^ Several approaches have been devised to circumvent these restrictions. In addition to semi‐quantitative morphological modifications,^26^, ^34^ techniques based on quantitative measurements have been commissioned, including t1rho,^35^ t2*,^36^ determination of T2 cutoff values,^20^ T2 mapping,^37^ and gagCEST.^38^ In addition, in conjunction with the development of MRI technology, more reliable results have been reported with the use of images obtained from devices with a higher field MRI (9.4 T) compared to devices with a field strength of 1.5–3 T.^24^ Kappa values between 0.46 and 0.94 were reported in the intra‐ and inter‐rater reliability tests performed in all these studies. Finally, the results of research studies employing AI networks have been published.^39^, ^40^ Despite claims that AI outperforms humans in classification, it cannot overcome classification's inherent limitations. So, none of these methods are readily available or simple to implement. Therefore, we anticipate that this qualitative morphologic classification with T2W sagittal sections, which can be easily obtained from any MR device, will be very useful in practical application.

The majority (36/42) of 42 discs with Pfirrmann stage 1 were found to be pre‐degenerative, or stage 2, according to the novel classification. With the new classification, only four discs remained in grade 1. We reckon that the utilization of VB signal intensity as a threshold made this distinction possible. These results indicate that 36 of the 42 discs have signal intensities below CSF but above VB. Intriguingly, while discs classified as Pfirrmann grade 1 were considered to be as hyperintense as CSF, the same discs were classified as grade 2 in 86% of cases and grade 1 in only 9% of cases by the same raters after the signal intensity of VB was accepted as a criterion in the new classification. In this instance, when VB intensity is not considered as a criterion, raters are able to match the brightness of the disc and CSF, whereas when VB intensity is taken into consideration, the majority of raters perceive that the disc intensity is not as hyperintense as CSF. This confirms previous research indicating that nucleus brightness correlates with proteoglycan density and not water or collagen content.^26^, ^41^ Increased heterogeneity was the sole distinction between Pfirrmann grade 2 and grade 1 discs; the presence or absence of clefts was not regarded as a distinguishing characteristic. We observed that grade 2 discs were distributed in the novel classification as 2‐3A‐3B. The brightness of VB, the increase in heterogeneity, and the presence of clefts, each of which is considered a distinct classifying feature, can be regarded as factors that contribute to this distribution in the novel classification. We observed that almost all of the discs in Pfirrmann grade 3 are distributed in grades 3A, 3B, 3C, 4A, and 4B, which are degenerative in the novel classification. Two of the discs in the grade 3 group were classified as predegenerative grade 2 (i.e., lighter gray than VB) and 6 were classified as grade 4 (black disc) based on the variations in gray shade by grade. With this discrimination, we observed that 10.8% of the discs shifted to grade 2‐4a‐4b. We anticipate that this rate reflects the patient group, which may change the choice of treatment. In contrast to Pfirmann, it is evident that the new system provides this distinction by arranging for the classification of morphological characteristics to evolve at each stage.

In the new classification, the majority of Pfirmann grade 4 discs fell into categories 3C–4A–4B. It is possible that the addition of disc margin irregularity as a new criterion, along with the partition of disc height reduction into two phases, contributes to the discriminatory power of the new classification. 16.1% of grade 4 discs were classified as grade 3b–3c in the new system. We think that this rate may indicate that the patient group may shift to conservative‐regenerative treatment instead of surgical treatment. The classification of five discs as grade 5 in the new system was based on the observation that the boundaries of the disc became completely irregular and collapsed. The classification criteria for grade 5 discs are similar in both classifications, but in the new classification, the criteria of complete disruption of the disc boundaries and contact of the vertebral bodies due to disc collapse have been added. In this group, we attribute the classification of some discs as grade 4B to the fact that, although the height loss was more than 50%, no border irregularity was observed. Therefore, we think that these features also increase the discriminative power of stage 5 discs. In this study, the Griffith classification was not used for comparison because the only distinguishing feature in the last four stages of the eight‐stage classification is disc height loss, which is categorized as 0–30%, 30–60%, and >60%. Particularly problematic appears to be the lack of external quantification for the differentiation of the 30%–60%‐classified grade 7 group from grades 6 and 8.

According to Binch et al., regenerative therapies should start in the early grades of degeneration, so detailed discrimination of patients with grades 2–3 in the Pfirmann classification will be a crucial factor in selecting the right patient at the right time.^19^ This discrimination may allow us to predict which patients will benefit from regenerative treatment as well as which patients will not; thus, it is possible to predict a socio‐economic benefit.

In addition to the advantages mentioned in terms of treatment guidance, we believe that this system can also serve as a guide to identify patients who may be candidates for quantitative classifications, which give very good results about degeneration by measuring various biochemical markers but have high costs and diverse application issues.

The main limitation of this study is that it has not been confirmed in clinical studies. This classification system has not been linked to the symptoms or treatment outcomes of patients. Nonetheless, this is a preliminary step toward assessing classification effectiveness in future studies based on both patient symptoms and treatment outcomes. Second, it has not been determined if it is associated with histological degeneration. Münarriz et al. previously reported that there was no correlation between Pfirrman scoring and histological Weiler's scoring in disc degeneration. The relationship between the novel eight class system and the degree of histological degeneration is another field of study.^42^

## 7 CONCLUSION

This study introduces an easy‐to‐use and discriminative MRI‐based classification system for lumbar disc degeneration and demonstrates its intra‐rater and inter‐rater reliability. This system may provide clinical and research benefits.

## AUTHOR CONTRIBUTIONS

*Concept*: Z.S. *Design of novel classification*, Z.S. *Supervision*: Z.S., E.B, D.U.U., A.C.I., C.S. *Materials*: Z.S., E.B., A.C.I., C.S. *Data collection and/or processing*: Z.S., E.B, D.U.U., A.C.I., C.S. *Analysis and/or interpretation*: Z.S., E.B, D.U.U., A.C.I., C.S. *Literature review*: Z.S., E.B., ACI. *Writing*: Z.S., E.B., DUU., ACI., C.S, *Critical review*: Z.S., E.B., D.U.U., ACI.

## CONFLICT OF INTEREST STATEMENT

The authors declare no conflicts of interest.

## INFORMED CONSENT

Informed consent was obtained from all individual participants included in the study.

## DECLARATION STATEMENT

All authors certify that they have no affiliations with or involvement in any organization or entity with any financial interest or non‐financial interest in the subject matter or materials discussed in this manuscript. The authors did not receive support from any organization for the submitted work. All authors have made substantial contributions in the interpretation of data, revising the article critically and all approved of the final version for submission. All authors disclosed no relevant relationships and conflict of interests.

## Supporting information


**Chart 1.** Supporting information.

## Data Availability

The data used during the current study are available from the corresponding author on reasonable request.
