# Supplementary material for: Tracing the disc: The novel qualitative morphometric MRI based disc degeneration classification system
Source: JOR Spine. 2024 Mar 18;7(1):e1321. doi: 10.1002/jsp2.1321 (PMC10945309; doi:10.1002/jsp2.1321)

GRADE 1: non degenerated

<u>Features:</u>

Uniform, homogenous density, normal disc height, hyperintense as or close to CSF, Nucleus Pulposus (NP) and Annulus Fibrosus (AF) clearly distinguishable.

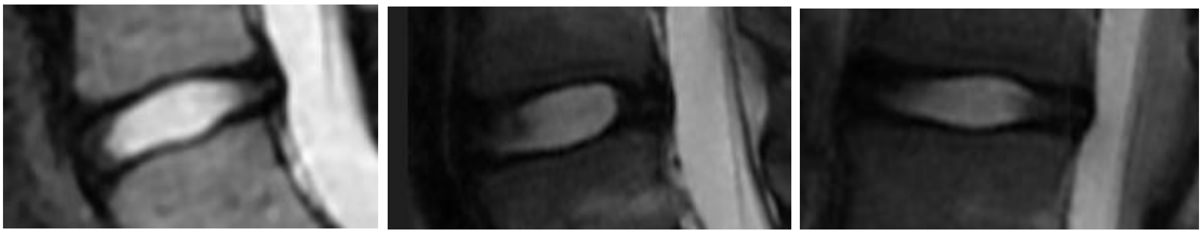

GRADE 2 : pre-degenerative

<u>Features :</u> Uniform, homogenous density, normal disc height, hypointense then CSF but hyperintense then vertebral body (VB).

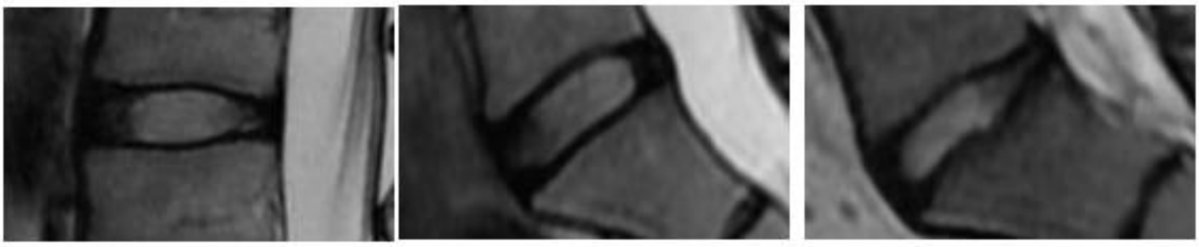

GRADE 3 : The Onset Of Degeneration, “gray disc”

<u>Features :</u> Homogeneity begins to disintegrate and heterogeneity initiates. The signal intensity of the disc is either equal to or commonly less than that of the vertebral body. The disc begins to turn gray. The height of the disc is largely maintained, and the distinction between the NP and AF remains distinct.

This stage is divided into three subsets.

A : Homogeneity is diminished, disc height is maintained, and there are no horizontal gray bands.

B : Heterogeneous appearance, disc height is still preserved and horizontal gray bands begin to appear.

C : Heterogenous, disc height begins to diminish, particularly posteriorly, there are diffuse dark gray signals in the disc and the distinction between NP and AF starts to disappear.

3A Samples, Homogeneity is decreasing but is still predominantly homogeneous; there are more gray signals than vertebral bodies, no HGB, and the disc height is preserved.

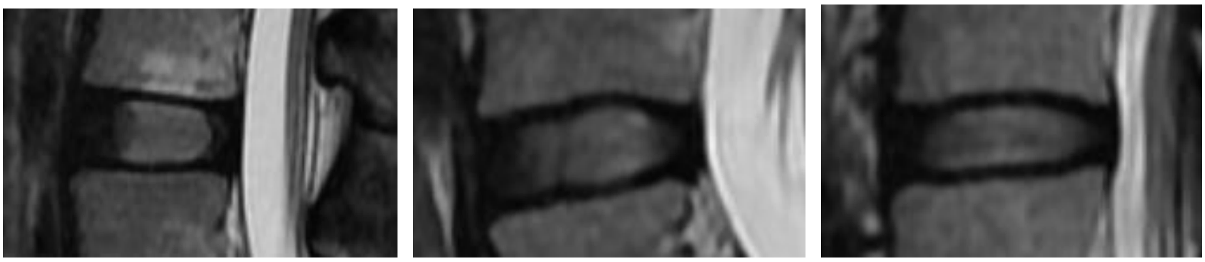

3B Samples, Homogeneity is further impaired. There are more gray signals from the vertebral body, the disc height is preserved, and horizontal bands become visible.

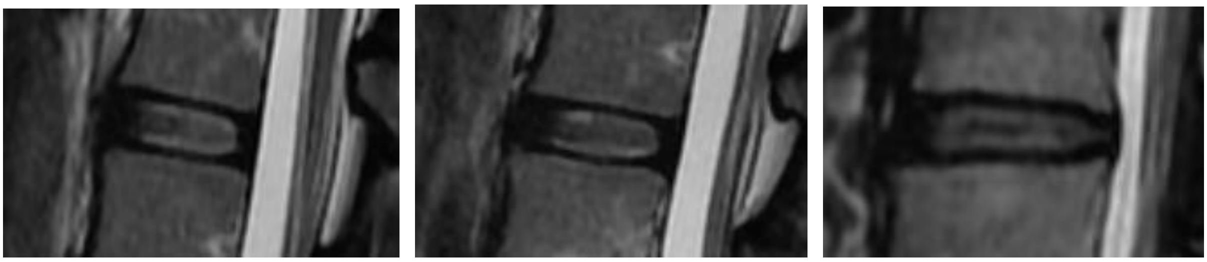

3C Samples: Signals become noticeably darker gray than in grades 3A–3B, with a loss of height, especially in the post. The distinguishing features are the loss of height and the darker gray. This darker gray signal distinguishes phase 3C from the previous two phases and can lead to confusion with the subsequent phase, grade 4. However, the primary point is that grade 3C is still gray, despite being darker.

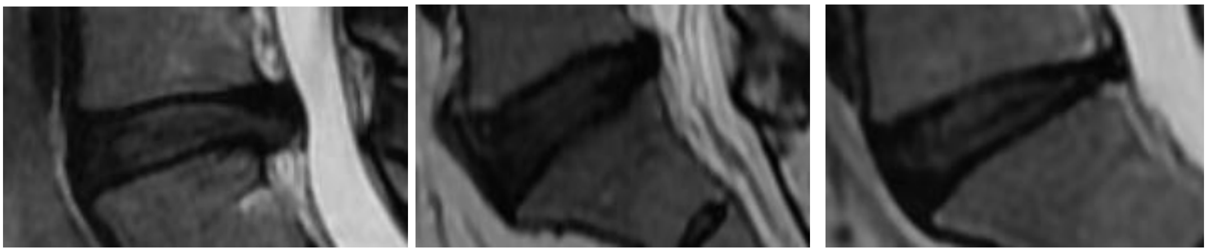

GRADE 4 : Degenerated, “black disk”

Hypointense **black disc**. Disc space began to diminish and become disorganized. The NP-AF distinction has almost completely disappeared.

A : Disc space is preserved by more than 50%, and disc borders are partially regular.

B : Disc space is reduced by more than 50%, and disc borders are irregular and bumpy.

4A Samples

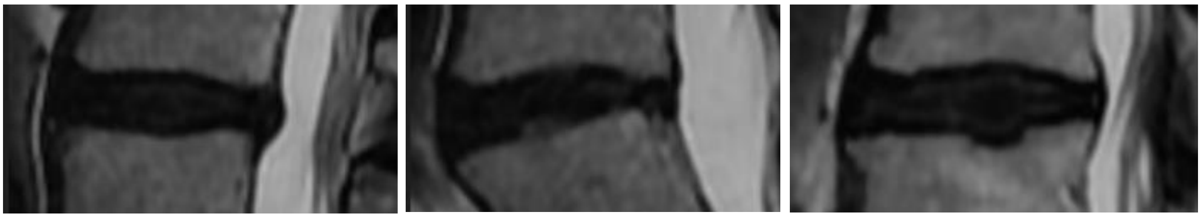

4B Samples

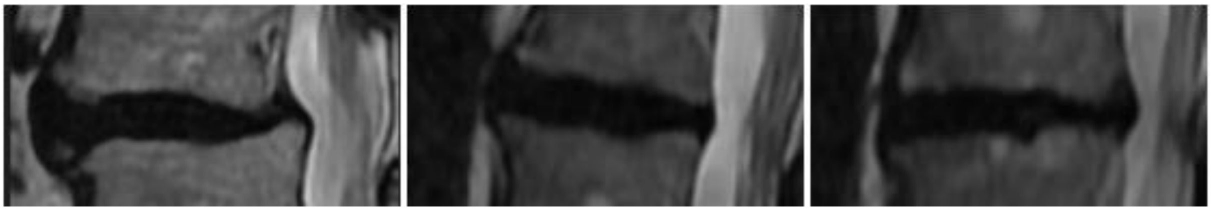

Grade 5 : Collapsed disk

Hypointense black signal. Disc borders have a bumpy appearance, and the NP-AF are indistinguishable. The disc is almost completely collapsed, and contact between adjacent vertebral bodies can be seen.

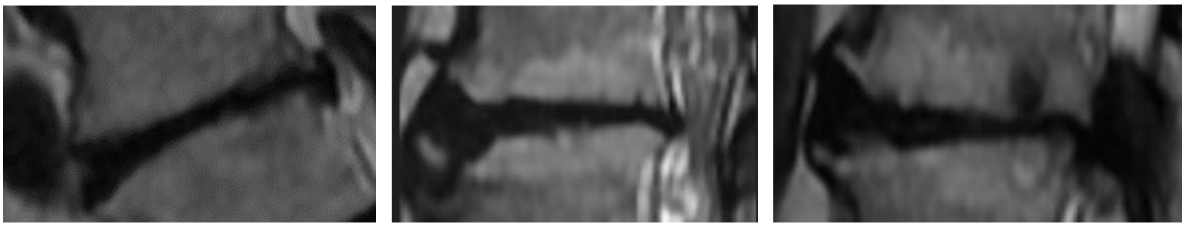

Supplement: Supplementary file 1 — Chart 1. Supporting information. [file JSP2-7-e1321-s001.pdf]
